# Construction of a model of endometritis in domestic rabbits using equine-derived pathogens and evaluation of therapeutic effect of sensitive drugs

**DOI:** 10.3389/fvets.2023.1064522

**Published:** 2023-02-09

**Authors:** Xuanpan Ding, Xiao Cui, Jinlian Shi, Xiaoli Cheng, Dan Yao, Yujin Gao, Yong Zhang

**Affiliations:** ^1^College of Veterinary Medicine, Gansu Agricultural University, Lanzhou, China; ^2^Gansu Key Laboratory of Animal Generational Physiology and Reproductive Regulation, Lanzhou, China

**Keywords:** horse, endometritis, animal models, inflammatory factors, NF-kB

## Abstract

Pathogenic bacteria were isolated from the uterine lavage fluid of a mare with endometritis. After identification and purification, the pathogenic bacteria were injected into the uterus of rabbits to induce endometritis. Then, anatomical, blood routine, chemical examination, and histopathological examinations were performed on the rabbits. Rabbit uterus was collected, and quantitative polymerase chain reaction (qPCR) was used to detect the mRNA expression of inflammatory factors including IL-1β, IL-6, and TNF-α in the rabbit uterus. In addition, enzyme-linked immunosorbent assay (ELISA) was used to detect the uterine concentrations of the inflammatory factors IL-1β, IL-6, and TNF-α. Western Blot was used to detect the protein expressions of NF-kB, IkBα, and TNF-α in the NF-kB pathway. An antibiotic treatment group was also set up to verify the accuracy of the results. The clinical examination results showed that there was a significant increase of leukocytes in the blood of the rabbits in the model group (*P* < 0.01). The uterus was congested, enlarged, and purulent. The integrity of the uterine lining was destroyed, and there was a significant increase of lymphocytes in the uterus (*P* < 0.01). The qPCR and ELISA results showed that the expressions of the inflammatory factors IL-1β, IL-6, and TNF-α in the uterus of rabbits were significantly increased (*P* < 0.01). Western blot results showed that the inflammatory factors IL-1β, IL-6, and TNF-α play a role in promoting inflammation through the NF-kB pathway. The results of the test provide a simple, economical, and reliable means of studying the occurrence, development, prevention, and treatment of equine endometritis.

## 1. Introduction

Equine endometritis is one of the common diseases in mares (1). The incidence of endometritis in infertile mares ranges from 25 to 60% (2, 3), which not only affects the normal physiological function of mares (4, 5), but also leads to mare reproductive disorders and even lifelong infertility (6–8). The disease is ranked in the third place (the first two are in turn the respiratory disease and gastrointestinal disease of horses) among the common diseases at the racecourse, causing greater damage to the breeding of horses (3). Bacterial infection is an important cause of equine endometritis. *Escherichia coli* and *Streptococcus equi* subsp *zooepidemicus* are the two most frequently detected pathogens of equine endometritis (8–10). For the treatment of this disease, the commonly used method is uterine irrigation combined with local and systemic antibiotics (7). Penicillin and streptomycin are the two commonly used drugs for the veterinary treatment of endometritis (10). Penicillin is a β-lactam antibiotic commonly used clinically for the treatment of Gram-positive bacterial infection, such as *Staphylococcus* and *Streptococcus*. Its pharmacological effect involves destroying the bacterial cell wall, thereby killing bacteria (11–13). Streptomycin is an aminoglycoside antibiotic, and its mechanism of action is to act on ribosomes in bacteria, inhibit bacterial protein synthesis, and destroy the integrity of bacterial cell membranes. Streptomycin has certain antibacterial activity against many gram-negative bacilli such as *E. coli* and *Klebsiella pneumoniae* (14, 15). In previous studies, it was found that some pathogenic bacteria had developed strong drug resistance (8). Currently, the emergence of drug resistance poses a serious challenge to the prevention and treatment of equine endometritis (3). In addition, due to the diversity of pathogenic bacteria and their subtypes, the development of vaccines is less obvious, and it is urgent to find new methods to prevent and treat this disease. Establishing the disease model is a simple and reliable to study the occurrence and development process of the disease and identify new treatment methods and drugs (16). However, in reality, horse is expensive, while a rabbit model has the advantages of low price, easy operation, and short experimental period (17). Therefore, rabbits were selected as experimental animals to establish the pathological model of equine endometritis and detect the relevant pathological indicators. Existing studies have shown that *IL-1*β, *IL-6, TNF-a*, and other factors play a crucial role in the occurrence and development of inflammation, which may lead to inflammation by activating the NF-kB signaling pathway (18–22).

## 2. Materials and methods

### 2.1. Animals

Eight mares with endometritis (Zhangye, Gansu) and 12 healthy adult female rabbits (2.5–3 kg, from Lanzhou Veterinary Institute, Chinese Academy of Agricultural Sciences) were used.

### 2.2. Isolation, identification, and amplification of pathogenic bacteria

We conducted clinical examination of mares that had been infertile for a long time on the farm and laboratory examination of mares suspected of having endometritis (fluid or swelling in the uterus upon examination) after rectal and ultrasound examinations. Briefly, the mare was bound, and the mare's vulva was disinfected using benzalkonium bromide (0.1% concentration). After disinfection, a double-layer sampling tube was inserted into the mare's vagina, and the inner tube was slowly inserted into the mare's uterus when the outer tube reached the cervix. Saline was injected into the uterus, and then the uterine flush was collected, followed by bacteria isolation and identification (see Supplementary material for results). The purified pathogens (*E. coli, S. equi* subsp *zooepidemicus*, and *S. aureus*) were inoculated into nutrient broth liquid medium containing sheep serum (Servicebio, Wuhan, China) and incubated for 12 h at 37°C on a shaker (23).

### 2.3. Establishment of the rabbit model of equine endometritis

According to previous research results on intraperitoneal injection of pathogenic bacteria in mice (Preliminary test—Figure 5 in Supplementary material), 10^9^ CFU/ml bacterial solution was selected for the modeling experiment. After 2 weeks of normal feeding, the rabbits were divided into control, model, and treatment groups (*n* = 4 per group). Adequate environmental disinfection (UV illumination) was done. First, the experimenter disinfected the rabbits' vulva using benzalkonium bromide (0.1%) according to the principles of aseptic operation. Then, 0.5 ml of *E. coli, S. equi* subsp *zooepidemicus*, and *S. aureus* were mixed and injected into the rabbit uteri of the experimental and treatment groups through a catheter, and the rabbits were lifted in reverse for 3 min after the injection to prevent the bacterial fluid from flowing out. The control group was injected with the same amount of nutritional broth. A total of three injections were administered per week (Days 1, 3, and 6). During this period, the rabbits were observed for body temperature, vaginal discharge, and other physiological conditions. One week later, the rabbits were dissected and observed for gross uterine lesions. After each inoculation, the rabbits in the treatment group were injected intramuscularly with penicillin (30,000 IU/kg) and streptomycin (10 mg/kg) according to the results of the previous drug sensitivity test against these pathogenic bacteria (Preliminary test—Tables 1–3 in Supplementary material).

### 2.4. Blood examination

Venous blood was collected from rabbits in the control, model, and treatment groups at the ear margins for routine blood analysis (MindrayBC-20s, Shanghai, China), with an emphasis on the changes in their white blood cells (WBC).

### 2.5. Chemical examination of uterine secretion and urine in rabbit

The uterine secretions were collected with a cotton swab, soaked in 2 ml of PBS solution, and an equal amount of sodium hydroxide (4%) was added. After mixing and observing, a colorless result is judged as negative, and a yellowish or lemon yellow result is judged as positive. Take 2 ml of rabbit urine, add 1 ml of silver nitrate solution (5%) and mix well, then heat to boiling, the appearance of black precipitate is positive, brown or light brown precipitate is negative.

### 2.6. Histopathological examination

Rabbit uterine tissues were fixed in 4% formaldehyde for 24 h. After paraffin embedding, sectioning, and hematoxylin-eosin staining (H&E staining), the morphological changes of the tissues were observed under a light microscope (APExBIO, Houston, USA) at a magnification of 100 × (24).

### 2.7. Immunohistochemical staining

The wax block-fixed uterine tissue sections (0.4 mm) were baked in an oven at 65°C for 2 h. The paraffin wax was melted and then removed with xylene. The sections were soaked in different concentrations of alcohol (100, 95, 85, 75%) for 5 min. The samples were removed, rinsed with distilled water for 3 min, rinsed with PBS for 5 min and then boiled with citrate solution, then cooked on low heat for 15 min, and after natural cooling, the sections were removed and rinsed with PBS for 5 min for antigen repair. The sections were incubated with H_2_O_2_ (3%) solution dropwise for 15 min for blocking endogenous peroxidase, rinsed with PBS for 5 min then antibody dilutions (IL-1β, IL-6, TNF-α and MPO; 1:200) were added dropwise and incubated for 12 h at 4°C. The incubated samples were removed and rinsed with PBS for 5 min, and then the secondary antibody dilution (IgG; 1:2,000) was added dropwise and incubated for 30 min at 37°C. The sections were rinsed with PBS for 5 min and then DAB color development solution was added dropwise. After color development, hematoxylin was added dropwise to re-stain the sections for 5 min. After rinsing with running water, alcohol was used to dehydrate the sections, xylene was used to clear the sections, and finally neutral resin was added dropwise to seal the sections. The fabricated sections were photographed and observed under a microscope at a magnification of 400 × . MPO and TNF-α were localized in the cell membrane or cytoplasm, IL-1β and IL-6 were localized in the cytoplasm. The proportion of positive cells in each high magnification field (400 × ) and the intensity of cell coloration were scored using the semi-quantitative integration method with reference to the scoring criteria of Fromowitz et al. (25). Color intensity score: no coloration was scored as 0, light yellow coloration was scored as 1, brown coloration was scored as 2, and tan coloration was scored as 3. The proportion of positive cells was scored: the proportion of positive cells < 1% was scored as 0, 1%−25% was scored as 1, 26%−50% was scored as 2, and >50% was scored as 3. The immunohistochemical score was obtained by adding the color intensity and the percentage of positive cells. The sum of the scores was 0–1, the expression was negative, with 1–2 being weakly positive (+), 3–4 being positive (++), and 5–6 being strongly positive (+++).

### 2.8. ELISA

Briefly, 0.1 g of uterine tissue was weighed in a mortar, liquid nitrogen was added, and the tissue was crushed. Then, the crushed tissue was mixed with 1 ml phosphate buffer saline (PBS), centrifuged at 3,000 rpm for 10 min at 4°C, and the supernatant was used for the assay. Next, 40 μl of sample diluent was added to the octal tube of the ELISA kit (JINGMEI BIOTECHNOLOGY, Jiangsu, China), mixed with 10 μl of supernatant sample, and incubated for 30 min at 37°C; the liquid was discarded, followed by washing with washing solution and pat drying. Then, 50 μl of horseradish peroxidase (HRP) was added to the wells, incubated for 30 min at 37°C, and the liquid was discarded, followed by pat drying on absorbent paper. Next, each well was filled with washing solution, washing was repeated five times, and then 50 μl of chromogen solution A and 50 μl of chromogen solution B was added to each well, followed by incubation at 37°C for 15 min. Finally, 50 μl of termination solution was added to each well, and the optical density (OD) of each well was measured at 450 nm using a microplate reader (Molecular devices, Shanghai, China). The concentrations of these three inflammatory factors were measured in rabbits using ELISA kits for IL-1β, IL-6, and TNF-α, respectively (26, 27).

### 2.9. Total RNA extraction and qPCR

TRIzol reagent (Servicebio, Wuhan, China) was used to extract total RNA from rabbit uterine tissue (28, 29). Briefly, 0.1 g of tissue was crushed and transferred to a centrifuge tube; 1 ml of TRIzol reagent was added and lysed at 4°C for 10 min; then, 200 μl of chloroform was added and mixed well. The mixture was allowed to stand for 10 min, followed by centrifuging at 12,000 rpm for 15 min. Next, 0.5 ml of the supernatant was mixed with an equal amount of isopropanol. After allowing to stand for 5 min, the RNA precipitate was obtained by centrifugation at 12,000 rpm for 15 min, and then dissolved in ddH_2_O after washing with anhydrous ethanol. The OD value (260/280) of RNA was measured using an Ultra Micro nucleic acid determinator (Pultton Technology, USA) and quantified to 200 ng/μl using ddH_2_O. RNA was reverse transcribed to cDNA using M-MLV reverse transcriptase. Then, 2 μl of 5 × gDNA clean reaction mix, 1 μl of RNA sample, and 7 μl of RNase free water were mixed and reacted at 42°C for 2 min to remove genomic DNA; then, 4 μl of 5 × EVO M-MLV RT reaction mix [contains RNase inhibitor, dNTPs, Oligo Dt(18T) primer, etc.] and 6 μl RNase free water was added and reacted at 37°C for 15 min, 85°C for 5 s, and then cooled to 4°C. The primers for β*-actin* (control), *IL-1*β, *IL-6*, and *TNF-*α were designed for rabbits (Table 1), synthesized (Tsingke Biotechnology, Beijing, China), and diluted to 10 μmol/ml. Then, 1 μl of cDNA was added to 10 μl of enzyme (2 × M5 Hiper SYBR Premix EsTaq), followed by 0.5 μl each of Forward primer and Reverse primer, and 8 μl of RNase free water. Pre-denaturing was performed at 95°C for 30 s, followed by 40 cycles of 95°C for 5 s and 60°C for 30 s. Results are expressed as mean relative quantification values, which were calculated using the 2^−Δ*ΔCT*^ method (30).

**Table 1:** Primer information.

**Cytokine**		**Primer sequence**	**Length of output (bp)**
IL-1B	NM_001082201.1	Forward primer: 5′-GGCCGATGGTCCCAATTACA-3′	243
		Reverse primer: 5′-TTGCAGAGGACGGGTTCTTC-3′	
TNF	NM_001082263.1	Forward primer: 5′-TCCTACGTGCTCCTCACTCA-3′	219
		Reverse primer: 5′-AAGGTCCAGGTACTCAGGCT-3′	
IL-6	NM_001082064.2	Forward primer: 5′-TGAGAATCACTTCGGGGCTG-3′	245
		Reverse primer: 5′-GTGGATCGTGGTCGTCTTCA-3′	
β-actin	NM_001101683.1	Forward primer: 5′-GTGAAGGTGACAGCAGTCGT-3′	145
		Reverse primer: 5′-GCTTCCGTCACATGGCATC-3′	

### 2.10. Determination of protein expression of inflammatory factors by Western blot

The protein content of inflammatory factors in the uterine tissue was determined by Western blotting (31, 32). Briefly, 0.1 g of uterine tissue was crushed in a mortar and poured into a centrifuge tube. Then, 1 ml of Radio-Immunoprecipitation Assay Buffer (RIPA Lysis Buffer, Servicebio, Wuhan, China) and 10 μl of the protease inhibitor Phenylmethylsulfonyl fluoride (PMSF, Abcam, Cambridge, UK) were added, followed by lysis for 30 min. Then, the samples were centrifuged at 10,000 rpm for 10 min at 4°C, and the supernatant was collected. The concentration was measured using the protein quantitative assay kit (BCA kit, JINGMEI BIOTECHNOLOGY, Jiangsu, China), protein loading buffer was added, and the samples were denatured at 95°C for 5 min. Polyacrylamide gel electrophoresis (12% SDS–PAGE) was performed on equal amounts of denatured protein samples, and the proteins in the gel were electrically transferred to polyvinylidene fluoride membrane (PVDF membrane, Servicebio, Wuhan, China). Then, the PVDF membrane was blocked in 5% skim milk for 2 h, and transferred to the diluent of the monoclonal antibody (Abcam, Cambridge, UK) of β-actin (control), IKBα, TNF-α, and NF-kB (antibody/antibody diluent = 1/1,000). The mixture was incubated at 4°C for 12 h, washed with phosphate buffer solution containing tween (PBST), and transferred to the secondary antibody diluent (Goat anti-mouse IgG, Abcam, Cambridge, UK). After incubation at 37°C for 2 h, PBST was used for washing, and ECL luminescence reagent (JINGMEI BIOTECHNOLOGY, Jiangsu, China) was added for exposure. The results were analyzed with Image J software (image J, Media Cybernetics Co. Rockville, MD, USA).

### 2.11. Statistics and analysis

The above trials were each conducted at least three times. Statistical analyses were performed using SPSS version 22.0 (SPSS Inc, Chicago, IL, USA). Data were analyzed by the *t*-test (between two groups). The statistical graphs were drawn using Prism version 5.0 (GraphPad software Inc, La Jolla, CA, USA). *P* < 0.05 was considered to indicate statistically significant differences.

## 3. Results

### 3.1. Obvious gross lesions

**Figure 1:**
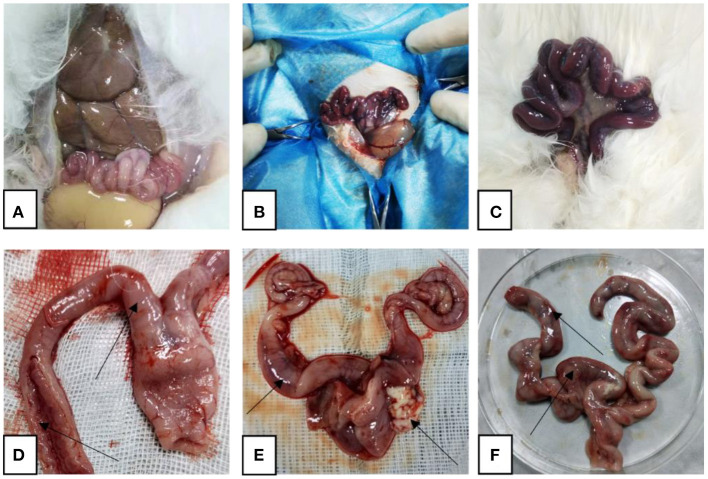
Representative gross autopsy pictures. **(A, D)** Control rabbit uterus; **(B, E)** model rabbit uterus; **(C, F)** treated rabbit uterus. Arrows point to: **(D)** uterus with normal shape and intact inner wall; **(E)** uterine hyperemia, pus outflow; **(F)** partial bleeding point.

After the rabbits were dissected, it was observed that the uterus of the rabbits in the control group was pink without symptoms such as congestion and swelling, while the uterus of the rabbits in the model group was dark red, congested, and swollen, and contained a large amount of pus. In the treatment group, the uterine congestion and swelling of the rabbits subsided (Figure 1).

### 3.2. Significant increase in blood WBC

**Figure 2:**
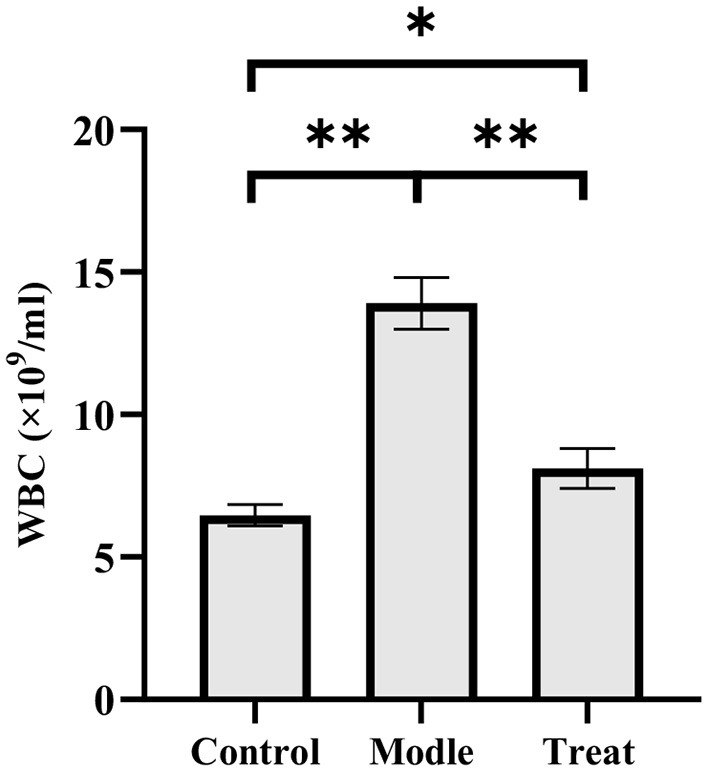
Examination results of white blood cells in rabbit blood. Bar graphs show the mean results ± SD (*n* = 3), *P < 0.05, **P < 0.01.

The blood routine results showed that rabbits in the model group had a significant increase in the number of WBC (*P* < 0.01). Compared with the model group, the number of WBC in the blood of the rabbits in the treatment group decreased significantly (*P* < 0.01), but it increased significantly compared with the control group (*P* < 0.05; Figure 2).

### 3.3. Positive biochemical test results

**Figure 3:**
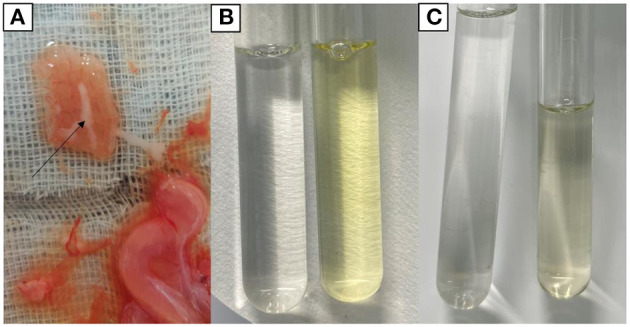
Chemical findings of uterine secretions. **(A)** Uterine secretions. **(B)** Control group (left) and model group (right). **(C)** Control group (left) and treatment group (right).

**Figure 4:**
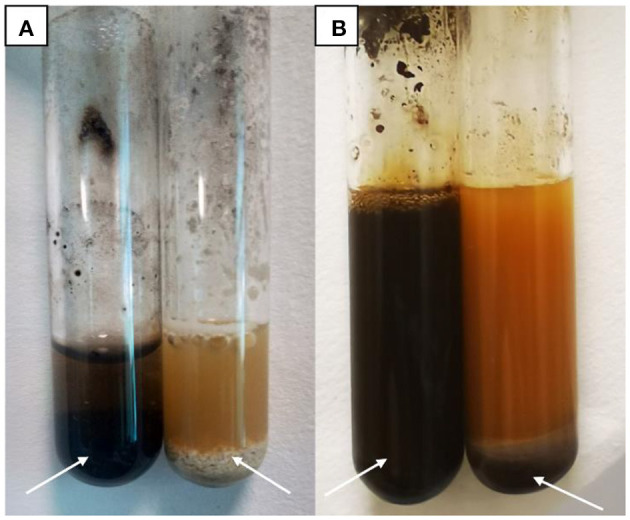
Urine Chemistry Test. **(A)** Model group (left) and control group (right). **(B)** Model group (left) and treatment group (right).

Chemical examination of uterine secretions showed colorless in the control group, yellow in the model group, and pale yellow in the treatment group (Figure 3). Urine chemistry showed brown deposits in the control group, a large amount of black deposits in the model group, and a small amount of black deposits in the treatment group (Figure 4).

### 3.4. Histological lesions of the uterus

**Figure 5:**
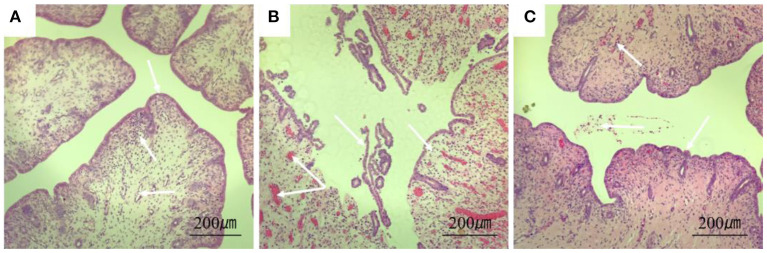
Section of rabbit uterine tissue (100×, H&E staining). **(A)** Rabbits in the control group; **(B)** rabbits in the model group; **(C)** rabbits in the treatment group. Arrows point to: endometrial cells, uterine vascular lumen, and lymphocytes.

The results of H&E showed that the endometrium of rabbits in the control group was complete, the structure was clear, and there were no obvious blood cells and lymphocytes in the stroma. In the model group, the endometrium was detached from the uterine body, had increased inflammatory exudates, and a large number of red blood cells were seen in the tissue. In the treatment group, a small amount of blood exuded in the stroma (Figure 5).

### 3.5. Overexpression of various inflammatory factors and MPO in the model group

**Figure 6:**
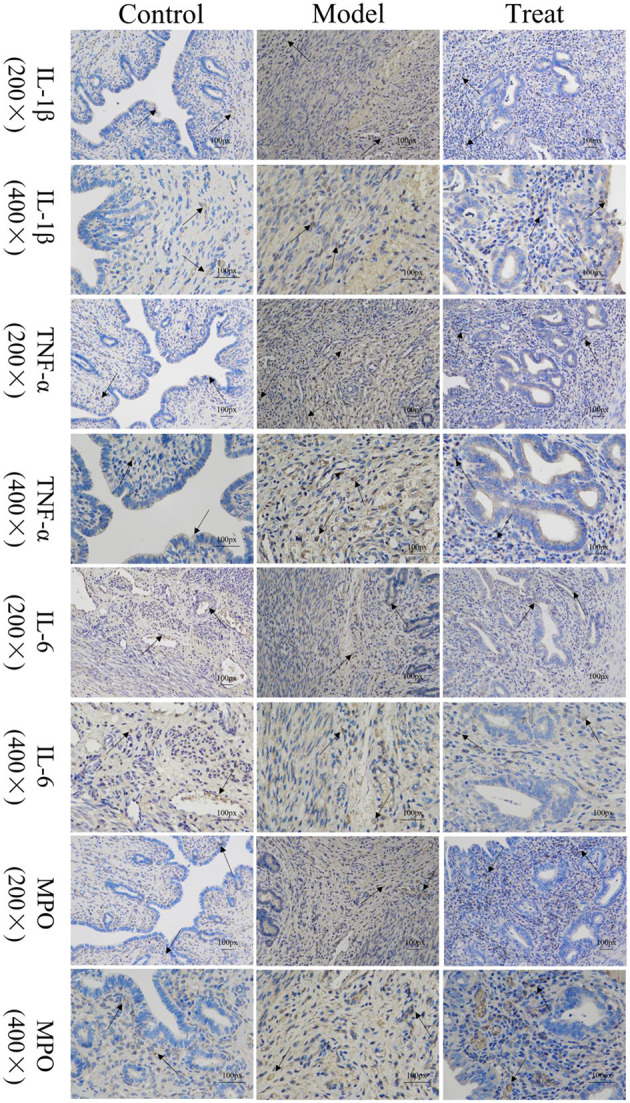
Localized expression of various inflammatory factors and MPO in uterine tissues. Arrows point to: positive staining area.

**Figure 7:**
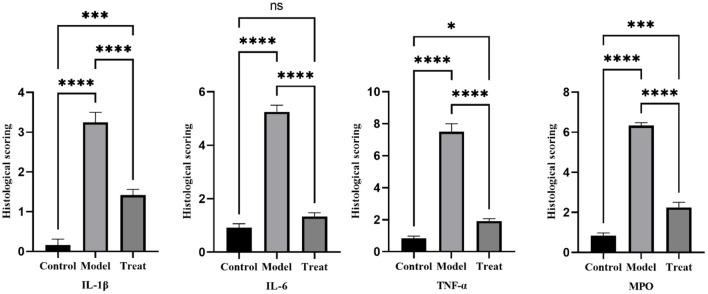
Histological scoring results. Bar graphs show the mean results ± SD (*n* = 3), * = 1: *P* < 0.05, *≥2: *P* < 0.01, ***P < 0.001, ****P < 0.0001.

Immunohistochemical results showed that the model group rabbits showed large amount of brown deposits in the uterus, the control group had basically no coloration, and the treatment group had a small amount of brown deposits (Figure 6). The mean scores of IL-1β, IL-6, TNF-α, and MPO in the uterus of control rabbits were 0.25, 1, 0.75, and 0.75, respectively, while the scores in the model group were 3.5, 5.25, 7.5, and 6.25, and the scores in the treatment group were 1.5, 1.25, 2, and 2.25. The expression of each factor in the model group was significantly increased compared with that in the control group (*P* < 0.01), and after the expression of each factor decreased significantly (*P* < 0.01) after treatment, but was still significantly higher (*P* < 0.05) than the control group except for IL-6 (Figure 7).

### 3.6. Altered concentration of inflammatory factors in the uterus

**Figure 8:**
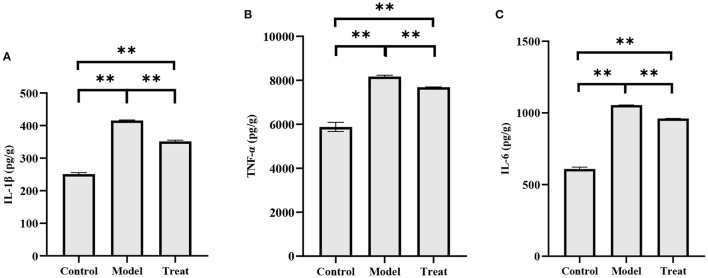
ELISA results. Bar graphs show the mean results ± SD (*n* = 3), **(A)** IL-1β; **(B)** TNF-α; **(C)** IL-6, **P < 0.01.

The concentrations of *IL-1*β, *IL-6*, and *TNF-*α in the uterus of rabbits in the model group were significantly increased (*P* < 0.01). Compared with the model group, the concentrations of *IL-1*β, *IL-6*, and *TNF-*α in the uterus of rabbits in the treatment group decreased significantly decreased (*P* < 0.01), but did not return to normal levels (Figure 8).

### 3.7. Differential mRNA expression of inflammatory factors in the uterus

**Figure 9:**
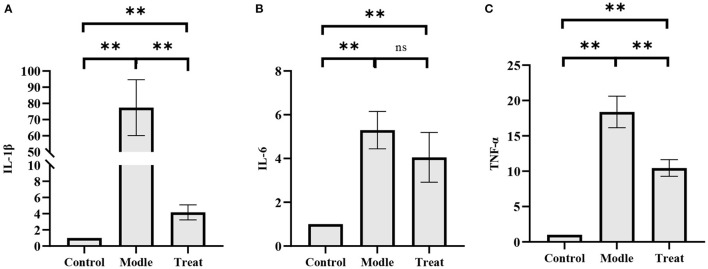
qPCR results. Bar graphs show the mean results ± SD (*n* = 3), **(A)** IL-1β; **(B)** IL-6; **(C)** TNF-α, **P < 0.01, ns: P > 0.05.

qPCR results showed that the mRNA expressions of *IL-1*β, *IL-6*, and *TNF-*α in the uterus of rabbits in the model group were significantly increased (*P* < 0.01). Compared with the model group, the expression of *IL-1*β and *TNF-*α in the treatment group was significantly decreased (*P* < 0.01), and the expression of *IL-6* was decreased, but the difference was not significant (*P* > 0.05; Figure 9).

### 3.8. Altered expression of inflammatory factors in proteins in the uterus

**Figure 10:**
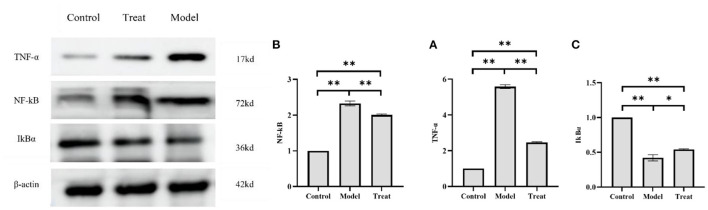
Western blot results. Bar graphs show the mean results ± SD (*n* = 3), **(A)** TNF-α; **(B)** NF-kB; **(C)** IkBα, *P < 0.05, **P < 0.01.

The results of western blot showed that the protein expressions of NF-kB and TNF-α in the uterus of rabbits in the model group were significantly increased (*P* < 0.01), and the expression of IkBα was significantly decreased (*P* < 0.01). Compared with the model group, the expressions of NF-kB and TNF-α in the rabbits in the treatment group were significantly decreased (*P* < 0.01), and the expression of IkBα was significantly increased (*P* < 0.05; Figure 10).

## 4. Discussion

Clinically, the causes of equine endometritis are diverse. However, bacterial infection is an important cause of acute equine endometritis (33). Lorenzo Pisello and Heather A. Davis conducted studies on the isolation and identification of pathogenic bacteria causing equine endometritis and the efficacy of antibiotics. The results found that the common pathogens of equine endometritis were *E. coli, S. equi* subsp *zooepidemicus*, and *Staphylococcus* (2, 3). In this experiment, the uterine lavage fluid of the mare with endometritis was collected (34), and three main pathogenic bacteria were found after bacterial isolation and identification, which were *E. coli, S. equi* subsp *zooepidemicus*, and *S. aureus*. This is consistent with the findings of Pisello et al. and Davis et al. The cultures of these three pathogenic bacteria were injected into the uterus of rabbits to explore the possibility of replicating equine endometritis in rabbits. After gross anatomy observation, uterine cytology, routine blood test, chemical examination and microscopic examination of uterine tissue sections, all the test results showed that the intrauterine injection of the pathogenic bacteria could induce inflammatory reactions in the uterus of rabbits. The experiment proved that it was feasible to establish a rabbit endometritis model with equine endometritis pathogens. Meanwhile, endometritis caused by bacterial infection usually involves inflammatory factors such as *IL-1*β, *IL-6*, and *TNF-*α. The concentrations and mRNA expressions of inflammatory factors (*IL-1*β, *IL-6, and TNF-*α) in rabbit uterine tissue were detected by ELISA and qPCR, respectively. The results showed that the expression of these three factors in the uterus of rabbits in the model group was significantly increased (*P* < 0.01). NF-kB signaling pathway is the main intracellular pathway of inflammatory response. Studies have shown that NF-kB signaling pathway participates in inflammatory response by inducing inflammatory factors such as *IL-6* and *TNF-*α (35–37). *TNF-*α is secreted by monocytes and macrophages. It is mainly involved in inflammatory cell signal transduction and induces the secretion of other cytokines, thereby triggering an immune response in the body. *IL-6* is synthesized by activated T cells and fibroblasts and plays a role in promoting inflammation in the inflammatory response. *IL-1*β is produced by activated macrophages and promotes the proliferation, differentiation, and function of cells involved in the immune response. After artificially injecting pathogenic bacteria into the uterus of rabbits, the pathogenic bacteria infection caused an inflammatory reaction in the uterus of rabbits, resulting in uterine congestion, swelling, pus accumulation, and other pathological manifestations (38, 39). After inflammation, the intrauterine environment of rabbits changes, causing apoptosis of uterine lining cells, which results in the destruction of endometrial integrity. Inflammation causes the body's immune response; therefore, white blood cells and lymphocytes in the blood and uterus increase. The invasion of pathogenic bacteria stimulates the immune response of the rabbit body, which triggers the production of inflammatory cytokines such as *IL-1*β, *IL-6, TNF-*α, activates IkBα kinase, and phosphorylates and degrades IkBα. The degradation of IkBα promotes NF-kB dimerization. In addition, the NF-kB canonical pathway is activated, which can result in a severe inflammatory response (35, 40–42).

Previously, some researchers successfully created a cow endometritis model with rabbits (43). However, the species of pathogenic bacteria of equine endometritis are quite different from those of bovine endometritis, possibly due to species differences. For example, *S. equi* subsp *zooepidemicus* has a high detection rate in equine endometritis but has not been reported in bovine endometritis. There is no report on whether rabbits can be used to create a model of equine endometritis. This study fills the gap and successfully created a pathological model of equine endometritis in rabbits. The expression of three inflammatory factors (*IL-1*β, *IL-6*, and *TNF-*α) in the model, and the mechanism involved in inflammation, the results show that these three inflammatory factors in the equine endometritis rabbit model. During the construction, the NF-kB signaling pathway played a role in promoting the occurrence of inflammation.

The inflammatory model was treated with penicillin and streptomycin. After observing the treatment effect, it was found that the use of antibiotics could significantly reduce the occurrence of inflammation, which was manifested by the decrease of lymphocytes in the uterus of rabbits; the return of endometrial cells to normal, and the return of rabbits' blood cells to normal (decreased number of white blood cells). Regarding the detection of inflammatory factors in the rabbits of the treatment group, it was found that the use of antibiotics can significantly reduce the concentration of each inflammatory factor and the expression of mRNA and protein. However, the emergence of drug resistance brings new challenges to the prevention and treatment of this disease. Thus, the construction of this model provides a simple and economical way to study the treatment of bacterial equine endometritis. However, with all the research methods used in the model, the effect of regression mares needs to be studied in more depth.

## 5. Conclusions

The rabbit model of endometritis was successfully established with the pathogens of equine endometritis, which provided a simple, economic, and reliable method for studying equine endometritis. In addition, our study also verified the effects of inflammatory cytokines (*IL-1*β*, IL-6*, and *TNF-*α) in the endometritis model. It was also found that activating the NF-kB signaling pathway the could promote the occurrence of inflammation.

## Data availability statement

The original contributions presented in the study are included in the article/Supplementary material, further inquiries can be directed to the corresponding author.

## Ethics statement

The animal study was reviewed and approved by Institutional Animal Care and Use Committee of Gansu Agricultural University (code GSAU-Eth-LST-2021-003). Written informed consent was obtained from the owners for the participation of their animals in this study.

## Author contributions

XCu and JS have made contributions to the isolation and identification of bacteria. DY, YG, and XCh participated in the construction of the pathological model and assisted in binding the test animals. XD designed and participated in the whole research and wrote this paper. YZ made great contributions to the design and completion of the experiment and the modification of the thesis. All authors contributed to the article and approved the submitted version.
