# Supplementary material for: Construction of a model of endometritis in domestic rabbits using equine-derived pathogens and evaluation of therapeutic effect of sensitive drugs
Source: Front Vet Sci. 2023 Feb 9;10:1064522. doi: 10.3389/fvets.2023.1064522 (PMC9948609; doi:10.3389/fvets.2023.1064522)
Supplement: Supplementary file 1 [file Data_Sheet_1.ZIP › Supplementary Materials/Preliminary test.docx]

The following information is preliminary to the trial, in which we carried out pathogen identification and pathogenicity testing and drug resistance analysis of equine endometritis.


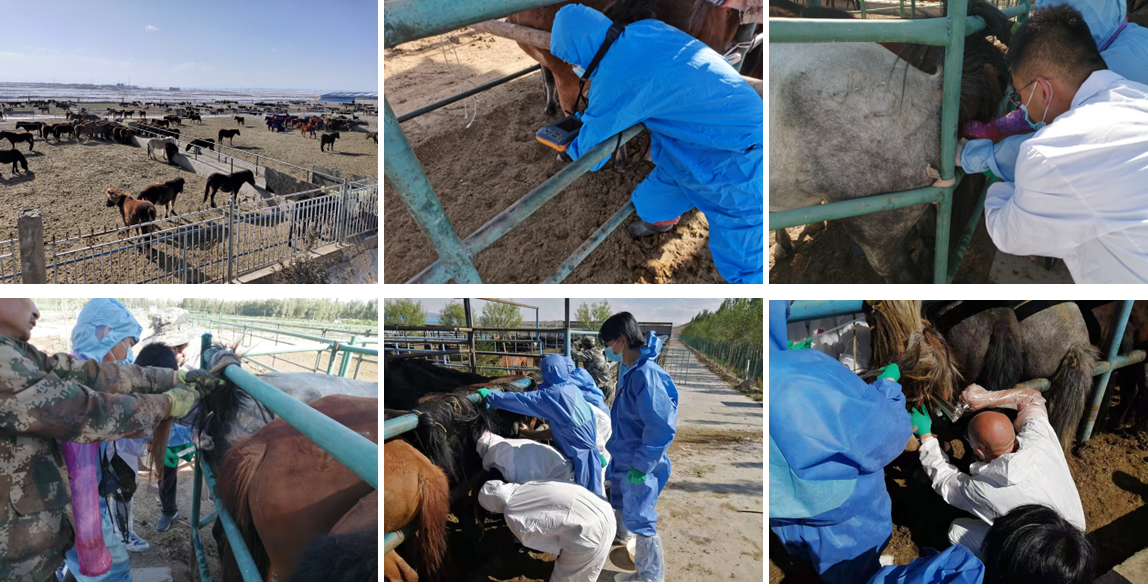


**Fig 1：**Clinical examinations such as B ultrasound are performed on mares suspected of suffering from endometritis, and the uterine irrigation solution of the mares is collected.


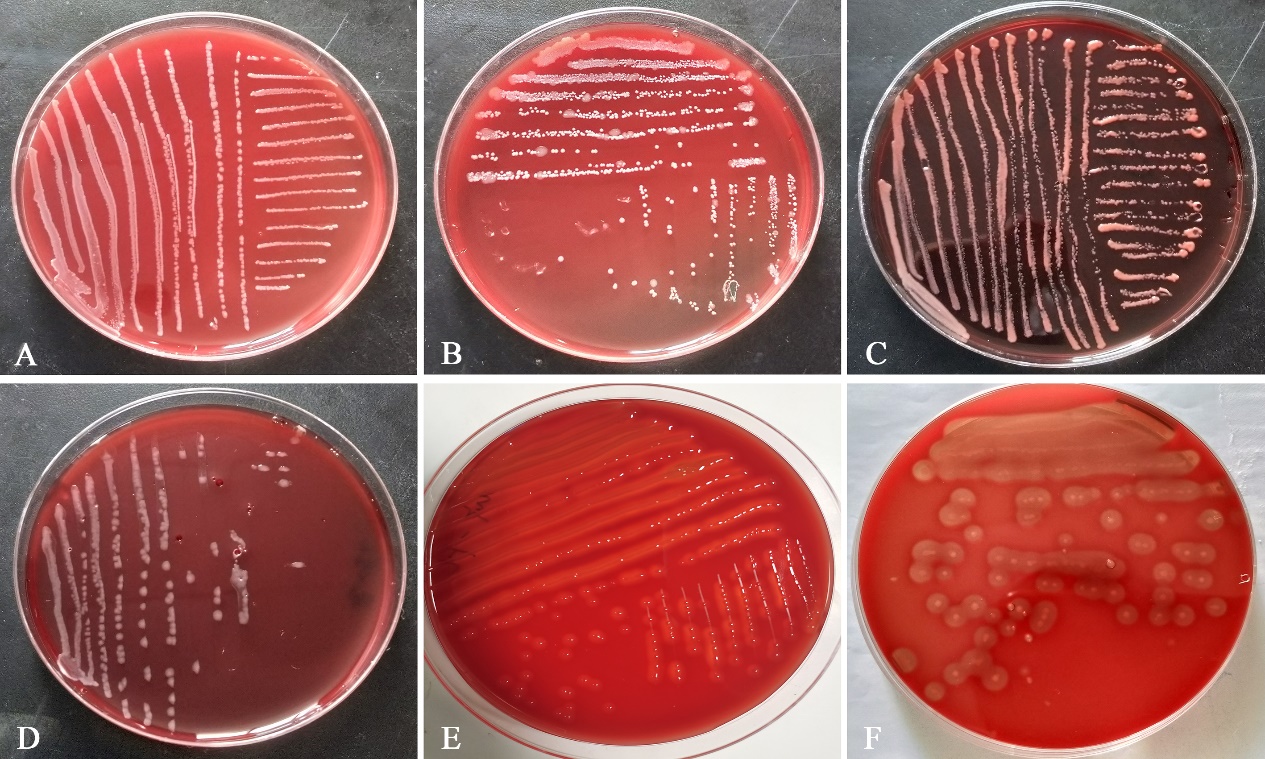


A:Escherichia coli; B:Staphylococcus; C:Streptococcus pneumoniae; D:Streptococcus agalactiae; E,F :s.equi subsp zooepidimicus

**Fig 2：**Growth morphology of isolated bacteria on 5% sheep blood agar plate


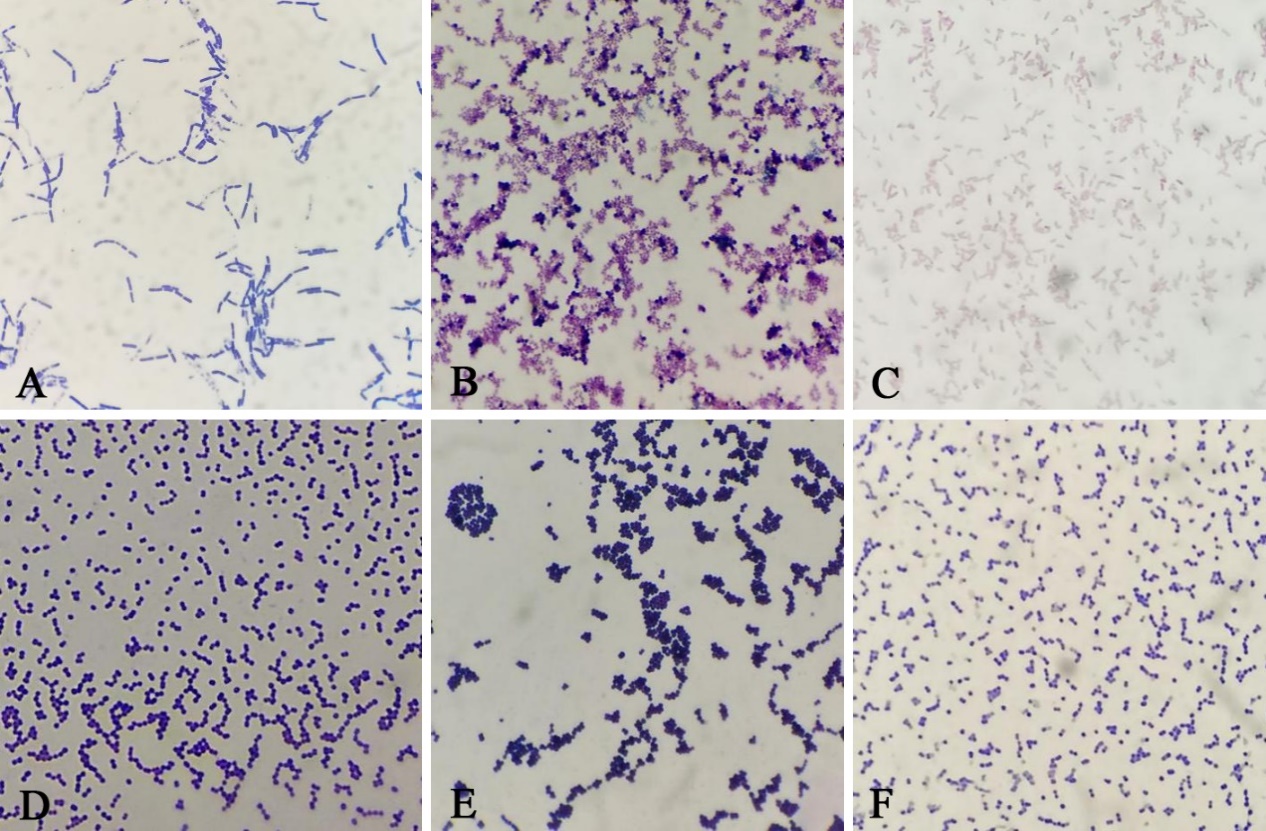


A:bacillus; B:Streptococcus pneumoniae; C:Escherichia coli; D:s.equi subsp. Zooepidimicus; E:Staphylococcus; F:Streptococcus agalactiae（×1000）

**Fig 3：**Microscopic examination of pathogenic bacteria (Gram staining)


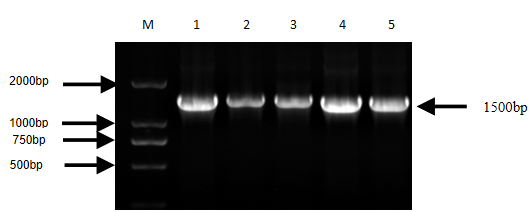


M: DL2000 DNA Marker; 1: Escherichia coli;2: s.equi subsp. Zooepidimicus ;3: Streptococcus pneumoniae; 4: Streptococcus agalactiae;5: Staphylococcus

**Fig 4：**16S rRNA electrophoresis results of pathogenic bacteria


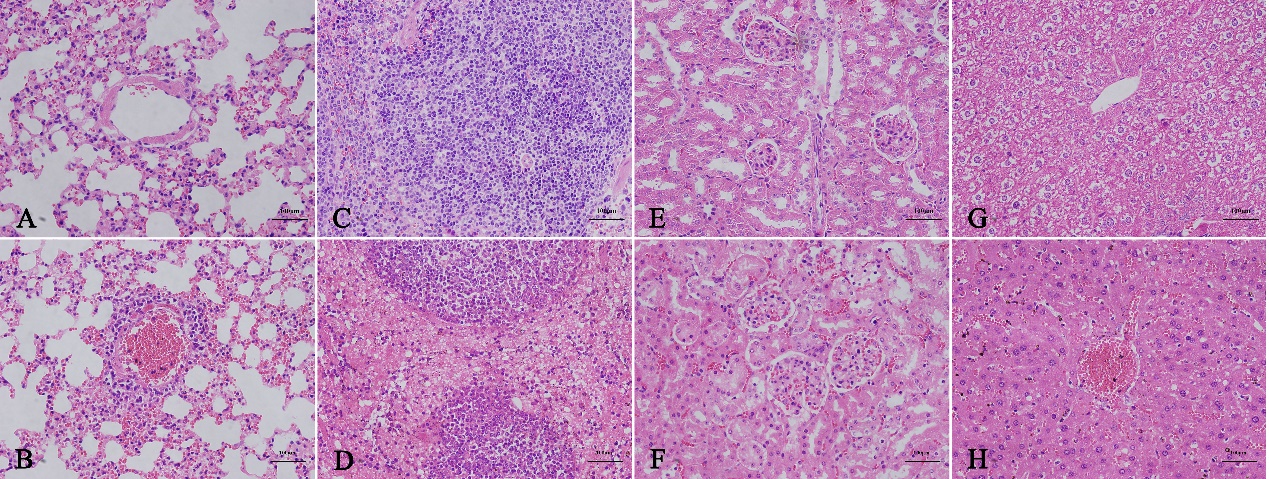


A: normal lung section B: pathological lung section C: normal spleen section D: pathological spleen section E: normal kidney section F: pathological kidney section G: normal liver section H: pathological liver section

**Fig 5:** Pathological tissue section of mice (HE staining, ×400)

**Table 1** Results of drug sensitivity test of *Escherichia coli*

|  | | | | | |
| --- | --- | --- | --- | --- | --- |
| Antibiotic | Antibacterial circle diameter (mm) | Criterion (mm) | | | Results |
|  |  | R | I | S |  |
| AMP | 17.08 | ≤13 | 14-16 | ≥17 | S |
| SCF | 28.06 | ≤15 | 16-20 | ≥21 | S |
| CTX | 25.21 | ≤14 | 15-22 | ≥23 | S |
| CZO | 20.59 | ≤14 | 15-17 | ≥18 | S |
| TE | 13.32 | ≤14 | 15-18 | ≥19 | I |
| CIP | 29.11 | ≤15 | 16-20 | ≥21 | S |
| STR | 21.31 | ≤11 | 12-14 | ≥19 | S |
| GEN | 14.68 | ≤12 | 13-14 | ≥15 | I |

**Table 2** Drug sensitivity test results of *Staphylococcus*

|  | | | | | |
| --- | --- | --- | --- | --- | --- |
| Antibiotic | Antibacterial circle diameter (mm) | Criterion (mm) | | | Results |
|  |  | R | I | S |  |
| E | 24.33 | ≤13 | 14-22 | ≥23 | S |
| P | 14.07 | ≤28 | - | ≥29 | R |
| AK | 21.02 | ≤14 | 15-16 | ≥17 | S |
| LEV | 29.11 | ≤15 | 16 | ≥17 | S |
| CZO | 31.95 | ≤14 | 15-17 | ≥18 | S |
| VA | 16.16 | - | - | ≥15 | S |
| SXT | 28.73 | ≤10 | 11-15 | ≥16 | S |
| GEN | 16.12 | ≤12 | 13-14 | ≥15 | S |
| OX | 27.72 | ≤10 | 11-12 | ≥13 | S |
| AMP | 18.31 | ≤28 | - | ≥29 | R |
| TE | 17.37 | ≤14 | 15-18 | ≥19 | I |
|  |  |  |  |  |  |

**Table 3** Drug sensitivity test results of *Streptococcus equi subspecies zoonotic*

|  | | | | | |
| --- | --- | --- | --- | --- | --- |
| Antibiotic | Antibacterial circle diameter (mm) | Criterion (mm) | | | Results |
|  |  | R | I | S |  |
| P | 36.71 | ≤19 | 20-25 | ≥26 | S |
| E | 37.28 | ≤15 | 16-20 | ≥21 | S |
| LEV | 26.84 | ≤13 | 14-16 | ≥17 | S |
| VA | 31.02 | - | - | ≥17 | S |
| TE | 25.11 | ≤18 | 19-22 | ≥23 | S |
| AMP | 26.28 | ≤18 | 19-28 | ≥29 | I |
| CTX | 46.97 | ≤25 | 26-27 | ≥28 | S |


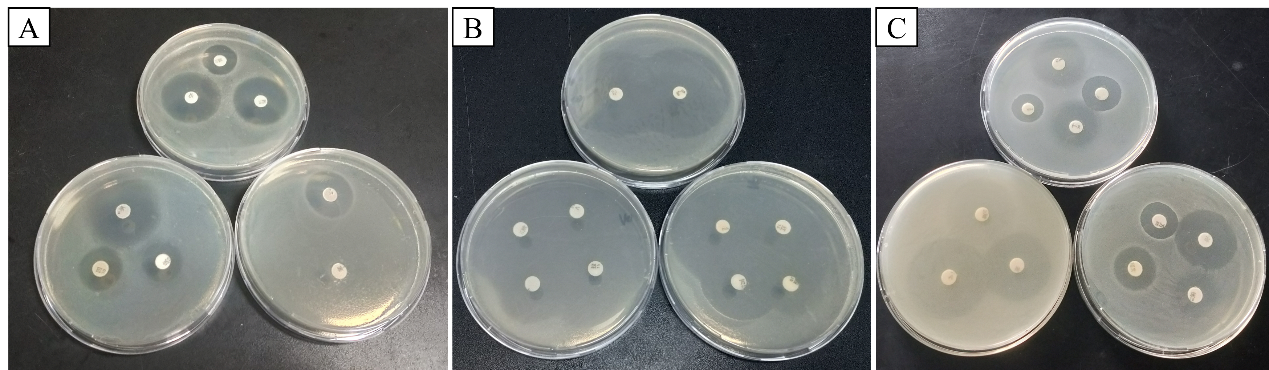


A:Escherichia coli; B:s.equi subsp. Zooepidimicus; C:Staphylococcus

**Fig 6:** Drug sensitivity test results


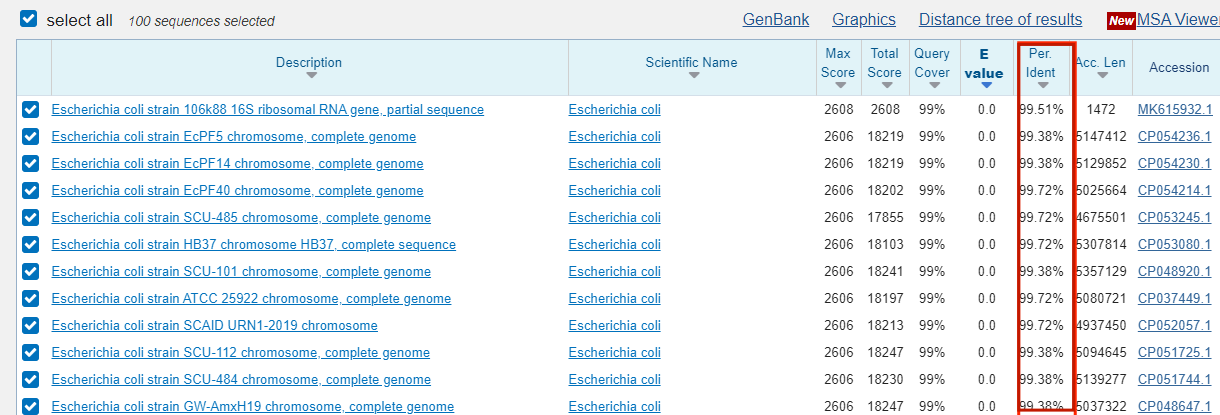


**Fig 7:** comparison results of *Escherichia coli*


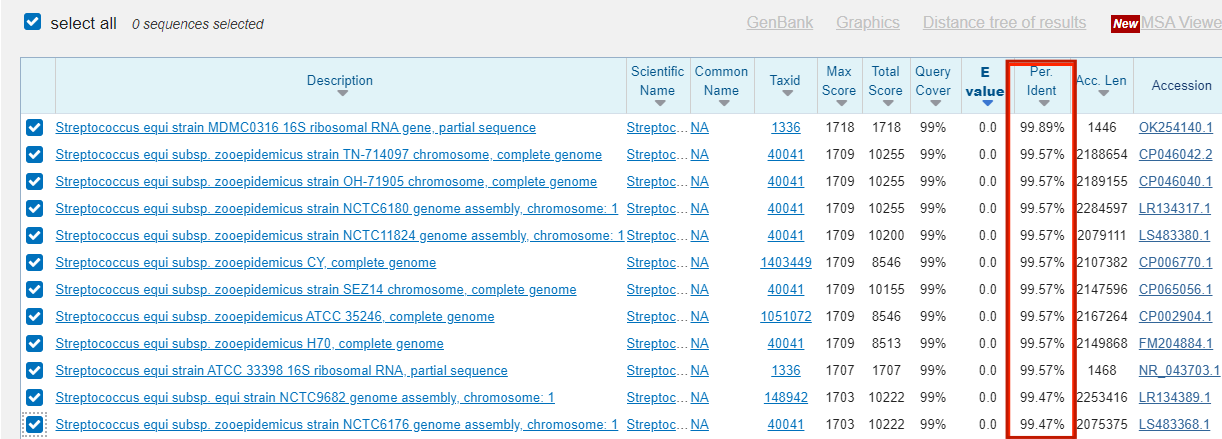


**Fig 8:** Comparison results of *Streptococcus equi*


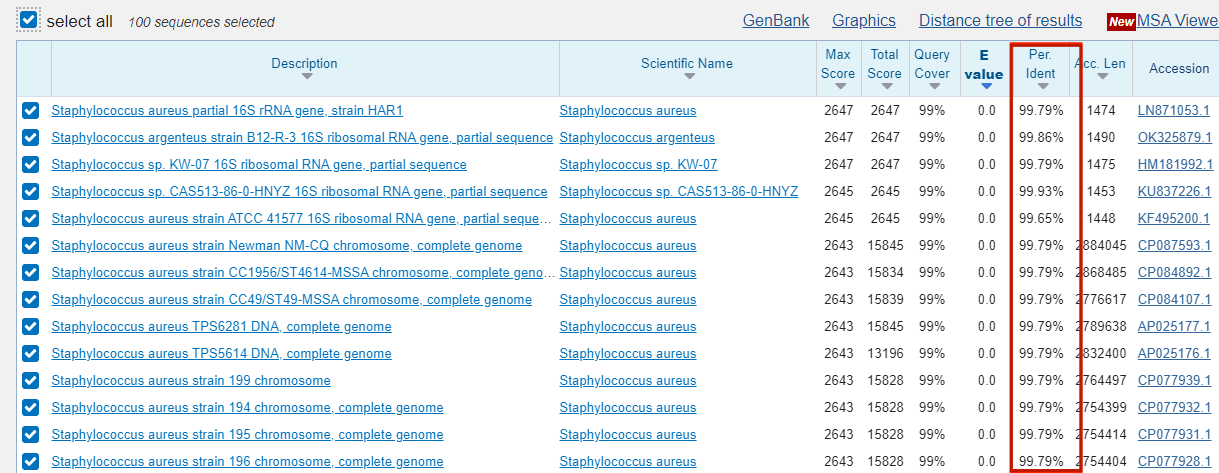


**Fig 9:** Comarison results of *Staphylococcus aureus*
